# BC-Monitor: Towards a Routinely Accessible Circulating Tumor DNA-Based Tool for Real-Time Monitoring Breast Cancer Progression and Treatment Effectiveness

**DOI:** 10.3390/cancers13143489

**Published:** 2021-07-12

**Authors:** Katalin Priskin, Sára Pólya, Lajos Pintér, Gábor Jaksa, Bernadett Csányi, Márton Zsolt Enyedi, Eszter Sági-Zsigmond, Farkas Sükösd, Orsolya Oláh-Németh, Gyöngyi Kelemen, Alíz Nikolényi, Gabriella Uhercsák, Dóra Sántha, Ágnes Dobi, Éva Szilágyi, Erzsébet Valicsek, László Tordai, Rozália Tóth, Zsuzsanna Kahán, Lajos Haracska

**Affiliations:** 1 Delta Bio 2000 Ltd., 6726 Szeged, Hungary; katalin.priskin@deltabio.eu (K.P.); lajos.pinter@deltabio.hu (L.P.); jaksa.gabor@deltagene.hu (G.J.); marton.enyedi@deltabio.eu (M.Z.E.); zsigmond.eszter@deltabio.hu (E.S.-Z.); 2 Visal Plus Ltd., 6726 Szeged, Hungary; sara.polya@visalplus.hu (S.P.); bernadett.csanyi@visalplus.hu (B.C.); 3 Department of Pathology, University of Szeged, 6701 Szeged, Hungary; sukosd.farkas@med.u-szeged.hu; 4 Department of Oncotherapy, University of Szeged, 6720 Szeged, Hungary; olah.orsolya@med.u-szeged.hu (O.O.-N.); kelemen.gyongyi@med.u-szeged.hu (G.K.); nikolenyi.aliz@med.u-szeged.hu (A.N.); uhercsak.gabriella@med.u-szeged.hu (G.U.); santha.dora@med.u-szeged.hu (D.S.); dobi.agnes@med.u-szeged.hu (Á.D.); szankane.szilagyi.eva@med.u-szeged.hu (É.S.); kosztane.valicsek.erzsebet@med.u-szeged.hu (E.V.); tordai.laszlo@med.u-szeged.hu (L.T.); toth.rozalia@med.u-szeged.hu (R.T.); kahan.zsuzsanna@med.u-szeged.hu (Z.K.); 5 HCEMM-BRC Mutagenesis and Carcinogenesis Research Group, Biological Research Centre, Institute of Genetics, 6726 Szeged, Hungary

**Keywords:** ctDNA treatment response, next-generation sequencing, liquid biopsy, breast cancer

## Abstract

**Simple Summary:**

Circulating tumor DNA (ctDNA) is increasingly employed in the management of malignant diseases, though the implementation of ctDNA diagnostics in daily oncology care has limitations. Here, we report our BC-monitor, a simple, well-balanced ctDNA diagnostic approach using an optimized multiplex PCR-based NGS protocol. We monitored a cohort of 45 breast cancer patients prospectively enrolled into our study receiving neoadjuvant chemotherapy, endocrine therapy, or palliative therapy for metastatic diseases. Their tumor mutation status was examined in the archived tumor samples and plasma samples collected before and during therapy. We detected traceable mutations in approximately two-thirds of the cases, and, importantly, new pathogenic variants in follow-up plasma that were not present in the primary tumor and baseline plasma. Our results indicate that ctDNA monitoring during treatment and follow-up with the BC-monitor tool, by predicting disease progression four–six months earlier than conventional methods and supporting treatment decision, may improve the outcome significantly.

**Abstract:**

Circulating tumor DNA (ctDNA) is increasingly employed in the screening, follow-up, and monitoring of the continuously evolving tumor; however, most ctDNA assays validated for clinical use cannot maintain the right balance between sensitivity, coverage, sample requirements, time, and cost. Here, we report our BC-monitor, a simple, well-balanced ctDNA diagnostic approach using a gene panel significant in breast cancer and an optimized multiplex PCR-based NGS protocol capable of identifying allele variant frequencies below 1% in cell-free plasma DNA. We monitored a cohort of 45 breast cancer patients prospectively enrolled into our study receiving neoadjuvant chemotherapy or endocrine therapy or palliative therapy for metastatic diseases. Their tumor mutation status was examined in the archived tumor samples and plasma samples collected before and continuously during therapy. Traceable mutations of the used 38-plex NGS assay were found in approximately two-thirds of the patients. Importantly, we detected new pathogenic variants in follow-up plasma samples that were not detected in the primary tumor and baseline plasma samples. We proved that the BC-monitor can pre-indicate disease progression four–six months earlier than conventional methods. Our study highlights the need for well-designed ctDNA monitoring during treatment and follow-up, integrated into a real-time treatment assessment, which could provide information on the active tumor DNA released into the blood.

## 1. Introduction

The demand for a long and healthy life increases, while the aging of the population puts serious pressure on the healthcare system and economy. With the advancement of laboratory diagnostics, in vitro diagnostic tests increasingly influence critical decision-making in medical treatments. When we think of diagnostics, the most important requirements are accuracy, speed, flexibility, and affordability. Due to improving cancer treatment strategies, many tumor types become long-term chronic conditions requiring continuous care [1]. Since a large number of publications have verified the applicability of free circulating tumor DNA (ctDNA) as a biomarker in the routine management of neoplastic diseases in the past few years, it can be foreseen that all oncological care facilities will require access to liquid biopsy in the near future [2,3,4]. Ideal diagnostics are characterized by high sensitivity while involving low health risks and costs in order to be repeatable and widely used.

Testing ctDNA requires a relatively noninvasive sampling; however, identifying mutations of low prevalence in heterogeneous samples with great accuracy is essential [5,6]. The first FDA-approved liquid biopsy test is hardly five years old and is utilized in EGFR mutation detection and the individualized therapy of NSCLC patients, especially in cases when, due to health conditions or the location of the tumor tissue, a biopsy cannot be performed [3]. The test is based on droplet digital PCR (ddPCR) technology, which provides a high sensitivity but has limited multiplexing capability. The FDA has recently approved the first next-generation sequencing (NGS)-based liquid biopsy test for treatment decision guidance [4]. The 54-gene ctDNA NGS test (Guardant360; Guardant Health, Redwood City, CA, USA), in contrast to analyzing a few variants in a determined genomic region one gene at a time, enables the simultaneous detection of mutations in frequent tumor genes in parallel.

Numerous clinical investigations (mainly in the metastatic setting) have relied on ctDNA genotyping, such as the BOLERO-2 study, the SOFEA and EFECT studies, the BEECH study, and the PALOMA-3 studies [5]. Fribbens et al. studied multiple genes with the ddPCR technique and NGS in serial samples of metastatic breast cancer patients on first-line aromatase inhibitor (AI) therapy. Subclonal mutations of the ESR1 (72%) and RAS genes (15%) were present in progressing patients [6]. Zhang et al. [7] performed NGS on the primary tumors and serial plasma samples (before and after surgery, after chemotherapy, and during the follow-up period) in 102 patients who needed chemotherapy after surgery. Using a highly sensitive method, about three-quarters of the cases had alterations in the plasma at the baseline, which could be followed later on in this high-risk population.

Considering the advantages and disadvantages of ddPCR and NGS-based ctDNA diagnoses is important when choosing one of these methods for a particular case. Digital PCR can monitor only a few hotspot mutations in parallel and cannot satisfy the requirement of simultaneously following many longer gene regions and hotspots from ctDNA. NGS technology enables the parallel investigation of large genomic regions and many genes; however, what we gain in the extended investigation of genomic regions, we may lose in detection sensitivity and cost. A more extended gene panel requires deeper sequencing, which increases costs. There are current technical limitations as well, since the error rates of standard Illumina NGS result in a critical decrease in the positive-predictive value at variant frequencies of ~1/100 and 1/1000 [8]. Nevertheless, the current stage of ctDNA NGS technology already provides clinicians with a better assessment of tumor composition and enables the selection of patients for mutation-directed therapies [9]. The more we learn about tumor evolution and treatment resistance, the higher the demand for ctDNA diagnostics will be. Diagnostic tissue sampling serves only as a mutational snapshot of the treatment-native cancer, and, during the course of the therapy, the mutational spectrum may change as the disease undergoes an evolution powered by both endogenous and exogenous factors, such as chemo- or radiotherapy, and endogenous mutational factors. Cancer evolution under selective pressure frequently leads to resistance-conferring genetic alterations that set back control over the disease. It has already been verified in metastatic breast cancer that ctDNA genetic contents vary over time during the therapy, mirroring the pharmacodynamic response to the targeted therapy, and while primary tumor samples and metastatic lesions can harbor different genetic profiles, all the primary tumor and metastatic alterations can be captured in longitudinal plasma monitoring [10].

Due to the benefits of flexible ctDNA technology in gaining a better understanding of cancer and the effects of medical interventions, a liquid biopsy could be utilized for staging and prognosis, the detection of minimal residual disease, or early relapse after primary therapy, as companion diagnostics for targeted therapy and the prediction of therapy response or resistance to it. In clinical investigations and drug development, the use of ctDNA technology seems essential [11]. Since we consider ctDNA technology a powerful biomarker assisting breast cancer management, we set out to test a self-developed NGS panel in a prospective pilot study of high-risk breast cancer patients in the environment of routine practice. Considering the above-mentioned limitations, an ideal ctDNA test method should have a gene panel focusing only on the most frequent aberrations in the given cancer, a minimized template requirement, tools for decreasing sequencing bias, and a reliable bioinformatic pipeline.

Here, we describe a widely applicable ctDNA screening and monitoring tool, BC-monitor, as a guide for breast cancer patient management in daily clinical care. Our ctDNA diagnostic approach relies on a gene panel specific to breast cancer, has a minimal template need, and is capable of identifying allele variant frequencies >0.5% in cell-free plasma DNA. To show its usefulness, we analyzed a cohort of 45 breast cancer patients prospectively enrolled and receiving neoadjuvant chemotherapy (NCT), neoadjuvant endocrine therapy (NET), or palliative therapy for metastatic diseases. The tumor mutation status was examined in archived tumor samples and plasma samples collected before and continuously during therapy. Clinical data, including CT images and the levels of the serum biomarkers CEA and CA-15-3, if available, were also collected. We found that BC-monitor was able to predict disease progression four–six months earlier than conventional methods and could potentially assist therapy decision-making via the real-time assessment of evolving mutations.

## 2. Materials and Methods

### 2.1. Design of the BC-Monitor Gene Panel

To develop a gene panel for ctDNA assay, we selected the most frequent genetic alterations in breast cancer that have predictive or prognostic value, guide targeted treatment, or underlie the most frequent resistance cases. First, we used The Cancer Genome Atlas (TCGA) [12] database to find significantly mutated genes in primary and metastatic breast cancer. Next, we prioritized genomic targets based on frequency and druggability. Lastly, we followed the ESMO Scale for Clinical Actionability of molecular Targets (ESCAT) classification system (https://www.esmo.org, accessed on 7 April 2021). We selected somatic variants from tier I and II that contain breast cancer-specific drug-matched alterations and supplemented them with TP53, one of the most frequent alterations in breast cancer, to increase the traceability of the tumor at the plasma level. The final list of targets (Table 1) was specified based on recent scientific and clinical literature highlighting the frequent somatic alterations in human breast cancer samples [13].

Our panel covered 38 genomic targets in 12 genes (AKT1, CDH1, EGFR, ESR1, FGFR1, FGFR4, HER2, HRAS, KRAS, MET, PIK3CA, and TP53). Primer design was performed manually, and all of the primers were checked for primer dimers and hairpin loops both with themselves and all other designed primers using the OligoAnalyzer web tool from IDT SciTools [14]. A universal tag and 12-nt-long random sequence (Unique Molecular Identifier, UMI) was attached to each forward and reverse primer 5′ end.

We checked our primers and used those that had minimum Hamming distance 3 (only mismatch, no indels) in the GRCh38-hg38 genome. We took care to avoid primer dimers and hybridization of the primers to each other, particularly at their 3′ ends.

### 2.2. Patients and Samples

This prospective cohort study was approved by the Institutional Ethics Review Board of the University of Szeged (#38/2018-SZTE), and all the enrolled patients gave their written informed consent before participation. Inclusion criteria were: (1) breast cancer needing neoadjuvant systemic therapy yet being anticancer therapy-naive (“neoadjuvant group”) or (2) breast cancer needing first-line systemic therapy for metastatic disease (“metastatic group”). Patients with any other malignant disease than breast cancer were excluded.

After enrollment, all patients were treated according to the institutional protocols, which were in consistence with the international guidelines. NCT was completed before surgery in all cases; postoperative radiotherapy and/or endocrine therapy were administered if needed. NET lasted for one year, and the same agent was administered postoperatively if, based on the pathology results, it was considered effective. Postoperative follow-up consisted of visits every six months that included yearly mammography and breast ultrasound but no other imaging studies in the absence of a suspicion of relapse. Ten milliliters of peripheral blood sample for ctDNA were taken at baseline, at halftime of the neoadjuvant therapy (typically two months and six months after the start of chemotherapy and endocrine therapy, respectively), before surgery, after surgery, and every six months thereafter.

Metastatic breast cancer patients received systemic therapies until progression or intolerable toxicity in a sequential manner. Restaging studies during the course of the disease did not differ from that of the usual clinical practice; the monitoring of circulating tumor markers such as CA 15-3 and CEA was not prescribed but was performed in many cases. Ten milliliters of peripheral blood sample for ctDNA determination were taken every three months or at the start of the next-line therapy.

Blood specimens were immediately centrifuged; separated plasma samples were stored in special cell-free DNA BCT collection tubes (Streck) at room temperature until processing.

Demographic data, tumor-related features such as the anatomical TNM stage and subtype, and the therapeutic modality applied were prospectively collected. Breast cancer subtypes were classified based on conventional immunohistochemical determinations (the cut-off value for ER or PR positivity was >10%) according to the St. Gallen consensus document as follows [15]:

luminal A-like (LUMA): ER- and PR-positive, HER2-negative, Ki-67 < 15%;
luminal B-like (LUMB): ER-positive, any PR, HER2-negative, Ki-67 ≥ 15%;
luminal B-like HER2-positive (LUMB HER+): ER-positive and/or PR-positive, HER2-positive, any Ki-67;
HER2-positive (HER2+): ER- and PR-negative, HER2-positive, any Ki-67;
triple-negative (TNBC): ER-, PR-negative, HER2-negative, any Ki-67.

Among the neoadjuvant cases, the therapeutic response to therapy was evaluated in the surgical specimen according to the Denkert tumor regression grade (TRG) system as follows: 0 = no effect, 1 = resorption and tumor sclerosis, 2 = minimal residual invasive tumor (<5 mm), 3 = residual noninvasive tumor only, 4 = no tumor detectable [16], and pathological complete response (pCR) if no invasive cancer tissue was found in either the breast tissue or the lymph nodes. Later on, the dates of eventual relapses were registered. Among the metastatic cases, therapy response was classified according to RECIST 1.1. Participation was terminated if the patient deceased or withdrew consent.

### 2.3. DNA Extraction and Quality Control

DNA was isolated from FFPE tissue samples using the MagCore Genomic DNA FFPE One-Step Kit (RBC Bioscience Corp. New Taipei City 23145, Taiwan), whereas cell-free DNA (cfDNA) from plasma samples was extracted using a NextPrep-Mag cfDNA kit (Bioo Scientific PerkinElmer, MA, USA), according to the manufacturer’s recommendations. Samples were then quantified by a Qubit^®^ 2.0 Fluorometer (Invitrogen, Milan, Italy) using a Qubit^®^ dsDNA HS Assay Kit (Life Technologies, Carlsbad, CA). If the concentration of the ctDNA isolate was less than 0.3 ng/µL, further concentration was carried out with the Agencourt AMPure XP system (Beckman Coulter, Inc., Indianapolis, IN, USA) (2× bead-to-sample volume ratio).

### 2.4. Barcoding and Library Construction

Multiplex amplification was performed in a reaction volume of 30 µL using 2 pmol of each primer, 0.6 U Q5 High-Fidelity DNA Polymerase (New England Biolabs, Ipswich, MA, USA), 1× Q5 Reaction Buffer, 1-mM MgCl_2_, 200 μM of each deoxynucleotide, and 5-ng cfDNA. The temperature profile was: 98 °C for 1 min, 25 cycles of 15 s at 98 °C, and 50 s at 62 °C. Primer ratios in the multiplex were optimized according to their coverage data on NGS.

Amplicons were purified using the Agencourt AMPure XP system (Beckman Coulter) according to the manufacturer’s instructions. The applied volume ratio between beads and PCR products was 0.8. The purified product was eluted in 15-μL AccuGene water (Lonza, Basel, Switzerland).

Indexing PCR was performed in 20 μL using 0.4 U Q5 High-Fidelity DNA Polymerase (New England BioLabs), 1× Q5 Reaction Buffer, 75 nM of each sequencing adapters, 200 μM of each deoxynucleotide, and 8-μL amplicon. The PCR conditions were as follows: 98 °C for 1 min, followed by 15 cycles of 15 s at 98 °C and 50 s at 63 °C. Illumina (https://www.illumina.com, accessed on 7 April 2021) Nextera XT sequencing adapters were extended with the sequence and the seven intermediate nucleotides. In addition, in order to increase the diversity in the amplicon library, each i5 sequencing adapter was used as a pool of four primers in which a ‘N’ (0–3) spacer was added between the Illumina sequencing primer and sequence.

Prior to sequencing, the dual-indexed library products were purified in the same manner as the amplicons and were quantified with a Qubit^®^ 2.0 Fluorometer (Invitrogen, Milan, Italy) using the Agilent High-Sensitivity DNA Kit (Life Technologies). DNA quality and correct sizing were checked by nested PCR using 5′-6-FAM-labeled primers for a fragment analysis on the 3500 Genetic Analyzer (Applied Biosystems Foster City, CA, USA).

The prepared libraries were sequenced on an Illumina NextSeq 550 System using the NextSeq 500/550 High or Mid-Output Kit v2.5 (300 cycles). Libraries were pooled at equimolar ratios for each run on the NextSeq 550 System and sequenced in paired-end mode (2 × 150 bp).

### 2.5. Data Processing

Individual reads were filtered for bases with low Q values with a maximum of 30 bases below Q28 allowed in the R1 and R2 directions for a read pair, resulting in 0.5–1% of the reads filtered out (filter level 1). Next, fastq read pair assembly was carried out with the modification that only the perfectly aligned read pairs were accepted, which was enabled by our primer design, resulting in products shorter than 285 base pairs [17]. With this tool, 33–50% of the reads were filtered out (filter level 2). Finally, based on the primer list, the primers were removed from the ends of the reads using the Smith–Waterman algorithm, allowing a 3-base Hamming distance, which is significant mainly in the case of overlapping primer pairs (filter level 3). Using the UMI sequences at each end, we clustered all reads with the same UMI and removed all read clusters that had more than one read (filter level 4) using UMI tools [18]. After the above filtering, the remaining reads were aligned to the GRCh38-hg38 genome using an algorithm based on Burrows–Wheeler Transform (BWT), keeping only the unique mappings.

### 2.6. Variant Calling

For variant detection, we included all bases above Q28 and listed all variants for the targets that were above a 0.15% frequency. For variant calling, we used freebayes v1.3.2 with the following parameters: min-alternate-fraction 0.0015—min-alternate-count 4—targets (our targets listed in BED format)—min-base-quality 28 [19]. All variants were annotated using SnpEff and validated manually by checking the alignment [20]; for the annotation, we used the transcripts from the MANE (Matched Annotation from the NCBI and EMBL-EBI) database using the onlyTr command line option. Coverage was calculated using the samtools bedcov tool (version 1.10); the BED file was created by including the middle nonoverlapping 1 bp in each target region [21]. Variants with frequencies between 0.15% and 0.5% were assumed true only if the matched tumor tissue also contained them. Clinical significance was annotated using SnpSift on the dbSNP and ClinVar variant databases [22]. If no annotation was present, we checked the available literature. Target coverage information was included for all samples where the whole panel was run and for samples from healthy individuals.

## 3. Results

### 3.1. Overview of the Breast Cancer-Specific ctDNA Diagnostic Tool (BC-Monitor)

**Figure 1:**
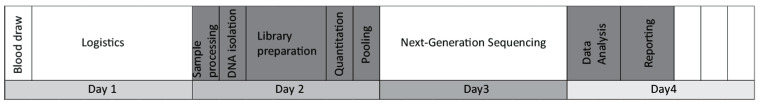
The BC-monitor workflow indicating the relative durations of the subtasks.

The main goal of this study was to evaluate the feasibility of a liquid biopsy-based NGS assay in the routine management of breast cancer. We named our self-developed test BC-monitor and outlined its workflow in Figure 1.

First, we established a multiplex PCR-based gene panel focusing on the most relevant variants in breast cancer regarding patient management. Primer design and sequence read processing were performed with our own in-house-developed pipeline. After setting up the PCR and NGS library preparation conditions, we estimated the accuracy and assay specificity using plasma DNA from healthy donors. To evaluate the utility of the assay, a clinical patient setting was included in the study, representing the most frequent breast cancer subtypes. All tumor tissue samples were analyzed using BC-monitor; however, the NCT and NET samples were collected prior to therapy, while the metastatic ones were collected before therapy changing from a core biopsy. A similar analysis for genotyping tumor and matched plasma samples was carried out using BC-monitor. By comparing the tissue and liquid biopsy results, we established a concordance between the changes in ctDNA and the course of the disease. By integrating the plasma follow-up data in the context of clinical care, we experienced the utility of our method in predicting disease progression earlier than conventional methods and providing information for a rational therapeutic choice.

In the first part of the laboratory assay development, we optimized the workflow from sample enrollment to reporting. Clinical data of the patients were systematically processed in an easy-to-use format and harmonized with the sequencing data. A standardized preanalytical protocol was used to ensure reliable cfDNA quality and quantity. Altogether, 293 plasma samples were processed in the study. During assay optimization, plasma samples were collected and stored at −80 °C after two rounds of centrifugation. The median hands-on time from blood drawing to report release was eight hours. Following the implementation of the panel into routine workflow for prospective samples, the average turnaround time was four working days.

### 3.2. ctDNA Monitoring NGS Assay and Bioinformatic Tool Optimalization

After optimizing the assay conditions using genomic DNA from healthy volunteers, we measured the specificity of the BC-monitor in 12 plasma cfDNA samples from healthy donors. The mean cfDNA concentration was 7.49 ng/mL of plasma (ranging from 2.8 to 14.2 ng/mL of plasma). We found no mutations in any of the control plasma samples, indicating a specificity of 100%. It must be noted that sequencing cfDNA from healthy donors only, without comparing it with DNA from normal white blood cells, is not enough to establish the real rate of the CHIP (Clonal hematopoiesis of indeterminate potential) phenomenon. 

**Figure 2:**
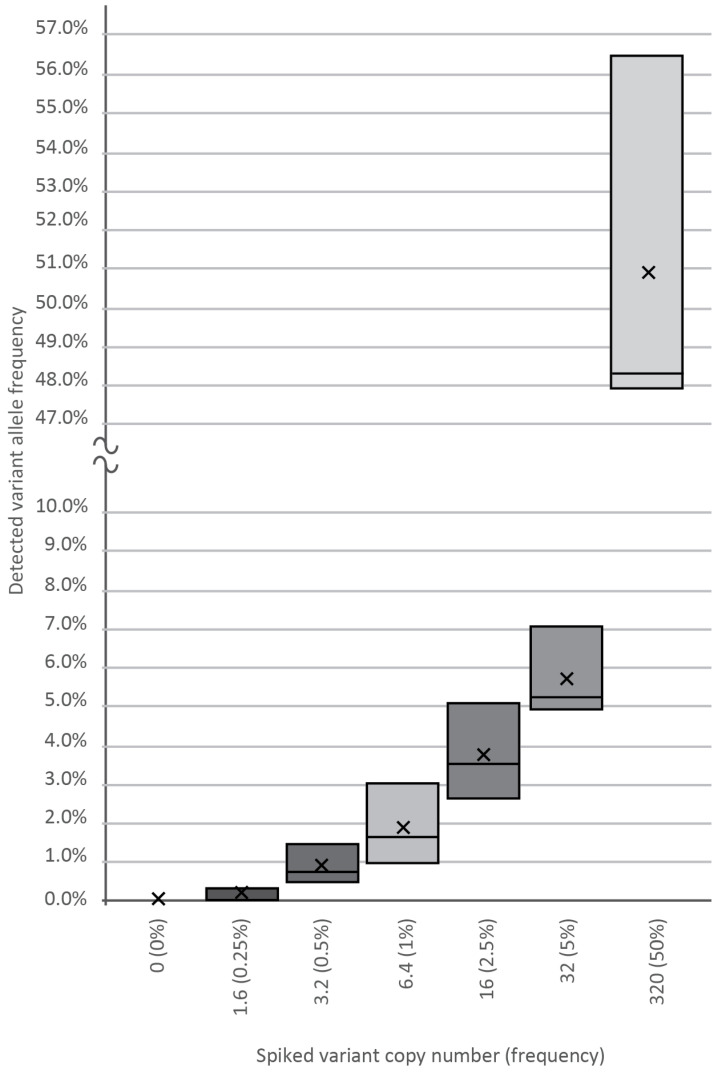
Sensitivity of the BC-monitor assay. Appropriate serial dilutions of cfDNA containing known SNPs were made, and each tube was spiked with 1.6, 3.2, 6.4, 16, 32, and 320 copies of the variant. Boxes indicate the interquartile range and median, while the average of each dataset is indicated by an X mark.

To determine the analytical sensitivity and reproducibility of our assay, cfDNA containing known SNP was spiked into wild-type plasma cfDNA samples at varying input amounts of 0, 1.6, 3.2, 6.4, 16, 32, and 320 template copies against a background of 640-WT DNA copies, as revealed by our previous genotyping. Plasma DNA from healthy volunteers served as a cfDNA source. After making amplicon libraries in three replicates, we generated a median of 10,000 NGS reads per spike-in sample, followed by variant calling and bioinformatics, which verified the frequency of the true mutant reads. As shown in Figure 2, using the BC-monitor method, we developed results with a high degree of correlation between the theoretical and measured variant frequencies of the serial dilutions.

To lower the effect of the sequencing bias, we filtered all reads on four levels (described in Section 2.5).

**Figure 3:**
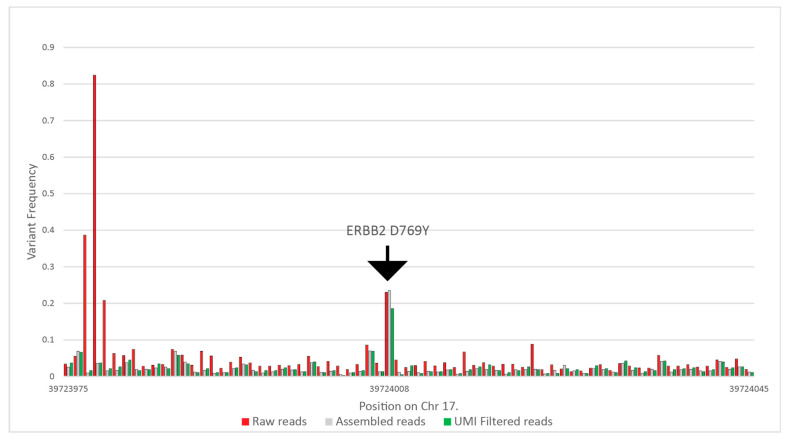
Calculated variant frequencies on the ERBB2 target and the surrounding nucleotides without filtering, using only the perfectly assembled read pairs, and eliminating all possible PCR duplicates using UMIs, from patient CT-72 at the 13th month of the study.

Raw reads were filtered for quality. In the case of each read pair, only those were included in the later steps that had a maximum of 30 bases below Q28; the number of bases was determined as 10% of the overall maximum read length. This step resulted in 0.5–1% of reads being excluded from the further steps. Next, we carried out an assembly of R1 and R2 reads, where only perfectly aligned reads were accepted to further lower sequencing errors on the overlapping regions. This step was enabled in the cases where we designed primers for shorter than 285-nt-long amplicons. The tool filtered out 33–50% of the reads. Subsequently, we tested the beginning and the end of each assembled read pair for the correct universal tag sequences with the Smith–Waterman algorithm, allowing two-base deviations, removing a further 1% to 2% of the reads. After that, using the UMI at each end, we clustered all reads with the same UMI and removed all read clusters that had more than one read. With this step, we removed all reads that were possible PCR duplicates. We included coverage information for samples where the whole panel was run, including the healthy samples as well in Table S1. Figure 3 summarizes the results of the above filtering on variant frequencies on a selected ERBB2 variant. In this particular case, the raw coverage was 434,449, which was filtered to 124,470 reads after assembly, and finally, 37,719 reads were used for variant calling.

### 3.3. Cohort and Patient Characteristics

**Figure 4:**
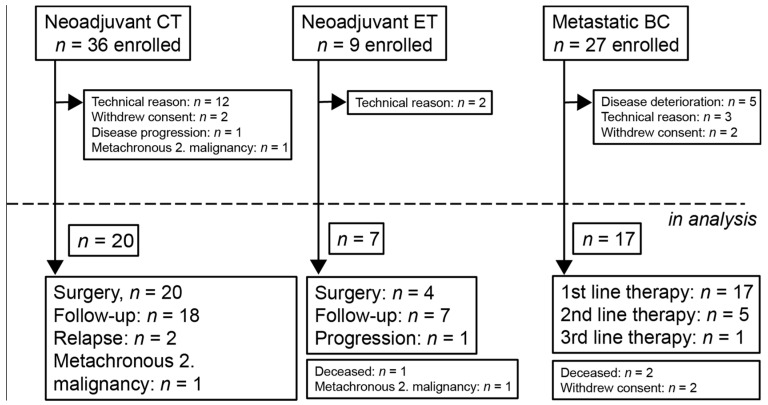
Patient flow diagram and possible reasons for dropping out according to treatment and setting. CT: chemotherapy, ET: endocrine therapy, and BC: breast cancer.

Between February 2018 and March 2021, 45 patients were altogether enrolled into the “neoadjuvant” (neoadjuvant chemotherapy—NCT; neoadjuvant endocrine therapy—NET) and 27 patients into the “metastatic” group (metastatic breast cancer—MBC) of breast cancer patients, and due to various reasons, 27 and 17 patients, respectively, remained in the analysis (Figure 4). The median follow-up times in the NCT, NET, and MBC groups were 21.2 (6.8–30.3), 23.2 (11.9–32.0), and 18.5 (1–33.7) months, respectively. In the NCT cohort, one patient received paclitaxel-carboplatin chemotherapy; all the other patients received taxane-anthracycline combinations. In four HER2-positive cases, chemotherapy was complemented with anti-HER2 therapy (trastuzumab ± pertuzumab). In all the NET cases, systemic therapy was an AI (letrozole or anastrozole) that was combined with goserelin in one premenopausal patient. Surgery was performed in all NCT patients but only in four of the NET patients; in three cases, surgery was refused by the patient. The treatment results, together with the individual tumor stages and subtypes of those neoadjuvant cases in which molecular aberrations were detected in the tumor or plasma, are included in Tables S1 and S2. Two of the NCT patients were not compliant in follow-up blood-sampling due to the COVID-19 pandemic situation.

**Figure 5:**
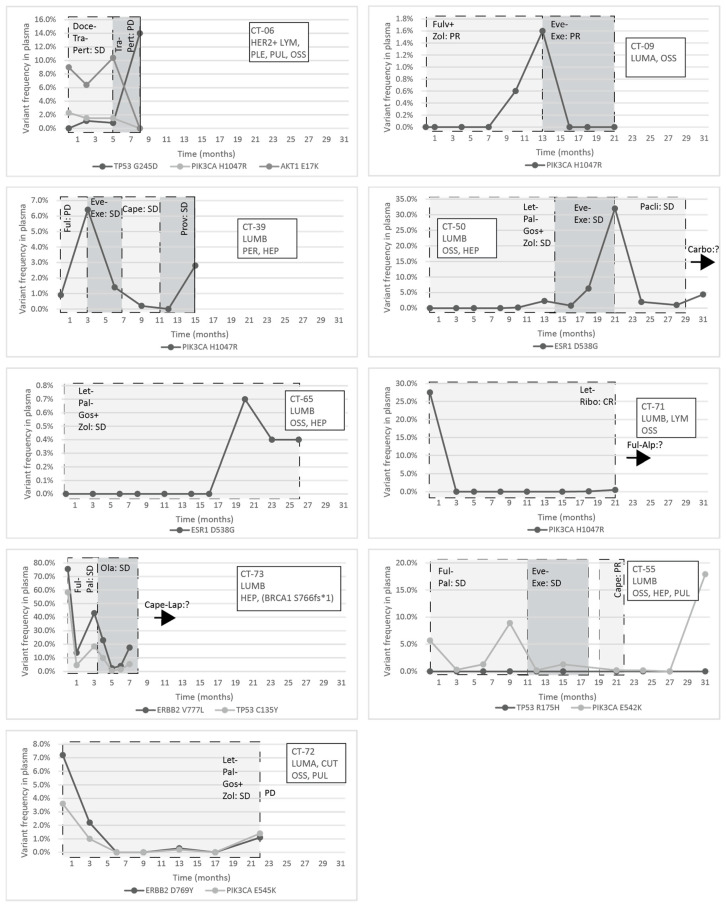
Circulating molecular aberrations in selected individual patients with metastatic breast cancer; the molecular subtype, metastatic organ involvement, the mutations detected in the primary tumor/metastasis, and the treatments and their effectiveness are indicated; the introduction of a new treatment modality was always due to progression, and the best response to a specific treatment is indicated within a column. If the perpendicular line is dashed, it indicates the change of a treatment modality, while, if contiguous, that means the loss of the patient. Abbreviations: LYM: lymph node metastasis, PLE: pleural metastasis, PUL: pulmonary metastasis, OSS: bone metastasis, HEP: liver metastasis, PER: peritoneal metastasis, CUT: cutaneous metastasis, CR: complete regression, PR: partial regression, SD: stable disease, PD: progressive disease, DTX: docetaxel, TRA: Trastuzumab, 2C4: Pertuzumab, LZL: Letrozole, PBB: Plabociclib, GOS: goserelin ZOL: Zoledronic Acid, EVL: Everolimus, EXM: Exemestane, PTX: Paclitaxel, CBDCA: Carboplatin, FUL: Fulvestrant, RBC: Ribociclib, ALP: Alpelisib, CAPE: Capecitabine, PRO: Provera, OLA: Olaparib, and LAP: Lapatinib.

Out of 27 enrolled metastatic breast cancer patients, 17 remained in the analysis (Figure 4). Individual data on the cancer subtypes, sites of metastases, and administered treatment modalities are included in Table S2 and Figure 5. Eleven patients received a single treatment modality throughout the study, while five patients were followed during two, and one patient during three, sequential treatment modalities.

### 3.4. Genotyping of Tumor Tissue and Plasma Samples

In total, 72 patients diagnosed with breast cancer were enrolled into the trial of the BC-monitor (Figure 4). Amplification failed in 12 FFPE samples due to low DNA quality. In four cases, due to the lack of FFPE samples, only previous diagnostic NGS data were used. In six cases, the baseline plasma sample did not contain enough cfDNA for downstream applications. Overall, 17 metastatic and 27 neoadjuvant patients were involved in tumor and serial plasma sequencing and the further analysis.

Altogether, 293 plasma samples were processed in the study, but only 258 samples contained adequate amounts of cfDNA. Initially, we used the 38-plex BC-monitor assay to genotype the FFPE tumor DNA obtained from the patients. Sequencing runs were carried out at a median coverage of 17,042 reads/amplicon after quality filtering. Results were assumed when at least 1000 reads were obtained for all target regions after filtering out low-quality reads.

Among the 27 cases in the neoadjuvant cohort, with FFPE tumor samples available, 15 had pathogenic mutations (55%), and in two cases, two mutations were detected; 8/15 patients (53.3%) had alterations in the PIK3CA, 7/15 (46.6%) in the TP53, and 1/15 (6.6%) in the AKT1 and ESR1 genes. In two patients, TP53 co-occurred with either PIK3CA or ESR1 aberrations. 

Out of the 14 FFPE DNA samples in the MBC cohort, 13 had at least one mutation (93%), while, in five cases, two mutations were detected. Among these, six (46.1%) had PIK3CA, eight (61.5%) TP53, three (23%) ERB2 mutations, and one (7.7%) aberration in both the AKT1 and ESR1 genes. In three patients, the ERBB2 mutation co-occurred with a further mutation—either TP53, PIK3CA, or ESR1—while TP53 and PIK3CA mutations also coexisted in one patient.

**Figure 6:**
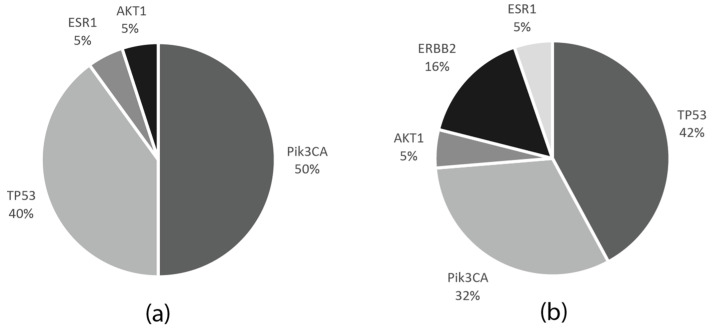
Frequencies of the gene mutations in the tumor samples of the metastatic and neoadjuvant patient cohorts. (**a**) Proportions of the mutated genes in the neoadjuvant (NCT and NET) patient cohorts. (**b**) Proportions of the mutated genes in the metastatic patient cohort.

PIK3CA mutations also coexisted in one patient. The frequencies of the mutations in the neoadjuvant and metastatic patient cohorts are indicated in Figure 6.

### 3.5. Concordance of Mutations in Tumor Tissue and Liquid Biopsy

The plasma samples (baseline and serial, *n* = 258) were also tested for mutations using the BC-monitor approach. For the metastatic cohort, baseline blood samples were taken at enrolment, and the patients underwent serial testing every three months, except for patient CT-73, who had a closer, monthly follow-up based on a clinical decision. The first plasma ctDNA analysis was performed at baseline (T0), before starting a new-line therapy.

In the NCT cohort, serial plasma samples were taken at baseline, during treatment, pre- and postoperatively, and then every six months during follow-up. Out of the 11 patients who had detectable mutations in the tumor, six patients had them in the baseline plasma. One patient did not have a tumor sample available, but the baseline plasma contained a PIK3CA mutation. Four patients harbored the same variant or variants in the plasma. In one case, out of the two mutations that were present in the tumor, only one could be detected in the plasma. One patient had a new mutation late in the follow-up period that was not detectable in the tumor tissue (Table S3).

In patients receiving NET, four out of seven harbored mutations in their tumor sample. From these, three patients had variants in the baseline plasma, and in the case of the fourth patient, the detected mutation appeared in the follow-up plasma sample. In one case, two variants were detected in the tumor, but only one out of the two were present in the baseline and the serial plasma samples. In one patient, the tumor did not contain a detectable mutation, but from the second plasma sample, a new mutation was detectable through the follow-up period (Table S4).

In four out of the 17 metastatic patients (23.5%), no mutation was detected in any of the follow-up plasma samples. In eight cases (47%), the same mutation or mutations were detectable in at least one of the serial plasma samples as in the tumor. One patient, who had only a TP53 mutation in the tumor tissue, carried two additional mutations in the serial plasma samples of the PIK3CA and AKT1 genes, suggesting subclonal heterogeneity in the tumor. Another patient acquired an ESR1 mutation, which was not detectable in the tumor or the first four plasma samples (Table S2).

There was no correlation between the total level of cfDNA and the rate of detection of the mutations.

### 3.6. Tracking Mutations in ctDNA: Monitoring Response to Therapy, Disease Course, Therapy Targets, and Resistance Mechanisms

In the NCT cohort, clinical progression was experienced in a single patient (CT-65); multiple bone, lung, and liver metastases were shown with CT in line with the clinical symptoms 8 months later; then, a newly developed TP53 R273C aberration was detected in the plasma.

In the NET population, one patient did not respond to anastrozole (CT-69): locoregional progression was observed in parallel with the increasing plasma level of ESR1 Y537C, while TP53 G245S also present in the tumor never occurred in the plasma. Although planned, due to noncompliance, the patient never received second-line therapy. In the CT-22 case, TP53 N239S was detected during NET 6 months before surgery. Although the clinical response to letrozole was poor, the patient was operated on, and since that, no progression of the breast cancer occurred. Nevertheless, a lung adenocarcinoma was diagnosed at the time of P2, which was surgically removed. No adjuvant therapy was given for that, while letrozole was maintained as the adjuvant therapy of the breast tumor. The specific TP53 mutation was not detected in the surgically removed lung cancer sample. Thereafter, the variant first decreased in the plasma (P3) but then progressively elevated. A local recurrence of the lung cancer was detected with PET/CT (at the time of P7). Definitive radiochemotherapy was applied.

In the MBC population of the patients, one actionable mutation—PIK3CA, ESR1, ERBB2, or AKT1—was found in ten patients and two in three cases either in the tumor or in the plasma. 

**Figure 7:**
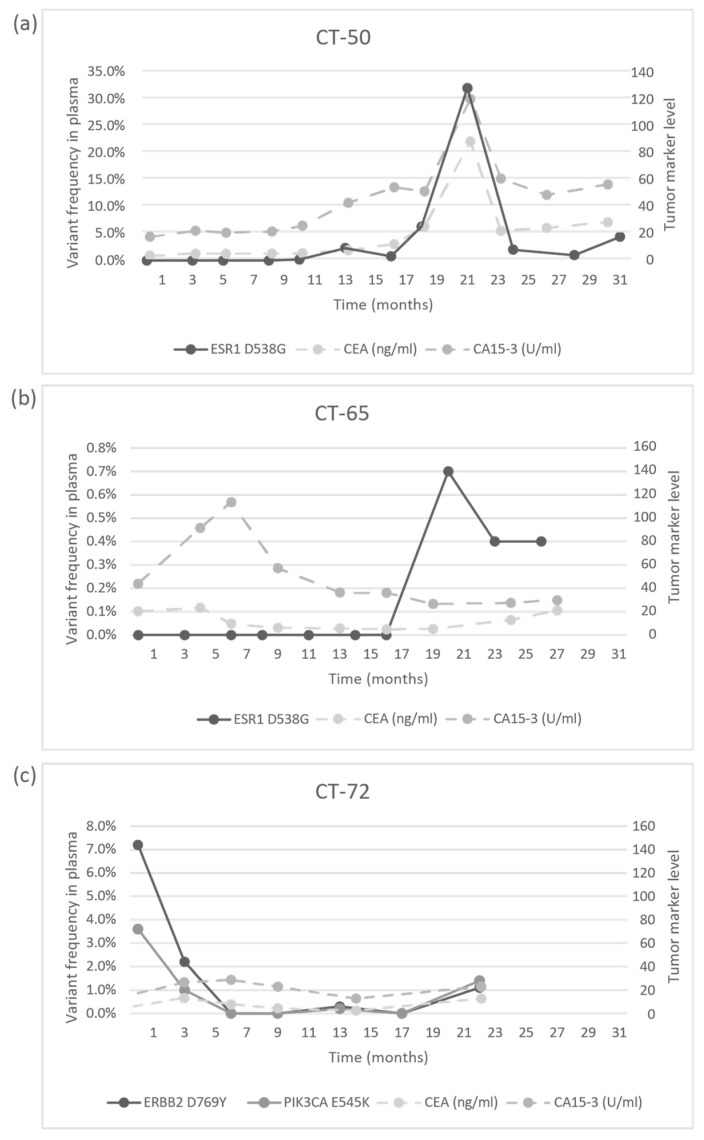
Circulating molecular aberrations in selected individual patients in relation to conventional tumor markers. (**a**) In the case of CT-50, CA 15-3 and CEA followed the level of tumor ESR1 D538G well. (**b**) In the case of CT-65, while CA 15-3 and CEA reflected well the favorable therapeutic response to letrozole-Palbociclib during the long treatment period, a newly developed mutation was detected with suspected disease progression and elevated tumor markers, but until the analysis, no clinical progression was validated. (**c**) In the case of CT-72, while the ctDNA alterations decreased following the start of treatment with letrozole-Palbociclib, the concentrations of CA 15-3 and CEA did not follow the tumor regression sensitively.

The response to treatment was evaluated with imaging studies and, in some cases, testing the circulating tumor markers CA 15-3 and CEA. In the CT-06 case, the termination of chemotherapy resulted in disease progression in parallel with the rise of the level of TP53 G245D possibly responsible for the resistance to anti-HER2 therapy. In the CT-09 case, the increase of circulating PIK3CA H1047R clearly predicted the evolving resistance to fulvestrant six months prior to clinical progression; bone scan and CT were performed at the time of clinical symptoms and tumor marker elevation, which showed new bone metastases; the specific everolimus-exemestane therapy caused a dramatic fall in its concentration; nevertheless, due to grade 3 treatment-specific pneumonitis, the patient refused further therapies (Figure 5). In the CT-73 case, we experienced very short treatment responses due to the presence of two resistance mutations (ERBB2 V777L and TP53 C135Y) and supposed mutational diversity related to the gBRCA1 mutation. In the CT-50 case, during Palbociclib-letrozole therapy, an acquired ESR1 mutation was detected 6 weeks earlier than true clinical progression was diagnosed by means of the CT, bone scan, and circulating tumor markers (note that the ESR1 mutation in the liver metastasis was detected at the time of disease progression, but in the primary tumor, no such alteration was verified); everolimus with exemestane was moderately effective, but chemotherapy could control the disease (note how sensitively ctDNA followed the disease status). In the CT-55 case, the PIK3CA E542K level decreased due to the treatment with Palbociclib-fulvestrant, but after nine months of therapy, its level increased again, predicting pulmonary, hepatic, and osseal progression that was detected two months later by means of a CT and bone scan; everolimus-exemestane was introduced, which controlled the alteration. In the CT-65 case, an ESR1 D538G mutation developed during the treatment with Palbociclib-letrozole raising the possibility of relapse 11 months earlier than the final analysis; interestingly, although circulating CA 15-3 and CEA were progressively elevated in parallel with the ctDNA variant, clinical progression could not be demonstrated by CT (although a bone scan indicated increased activity in the already detected bone lesions); if progression was verified, fulvestrant was planned as a next-line therapy. In the CT-71 case, after 15 months of ribociclib-letrozole therapy resulting in the CR of the lymph node and SD of bone metastases, a slow elevation of PIK3CA H1047R occurred; this aberration could guide the therapy decision: when the progression of bone metastases was verified by means of a CT, bone scan, and tumor markers 3 months later, alpelisib-fulvestrant therapy was started. In the CT-72 case, the two gene aberrations in the plasma frequently related to treatment resistance were well-suppressed by Palbociclib-letrozole therapy but started to rise again after 17 months of therapy; the patient was a candidate for alpelisib-fulvestrant therapy (Figure 5). Tumor markers rarely reflected the ctDNA changes but, rather, indicated the cancer load (Figure 7).

## 4. Discussion

Recent studies have verified the unambiguous role of ctDNA analysis in the genetic characterization of tumors and in the detection of preclinical relapses or metastases. Since long-term therapy can dynamically change intratumoral and intertumoral heterogeneity, there is an increasing demand for the continuous monitoring of the genetic changes during the entire disease continuum. For this purpose, ctDNA seems advantageous, since it reflects the continuously evolving mutational landscape of the primary and recurrent tumors. Thus, the implementation of ctDNA diagnostics in daily oncology care seems rational.

An ideal ctDNA diagnostic gene panel should focus on the most frequent aberrations in the given cancer type, thus minimizing the amount of required template and sequencing reads, as well as bioinformatics capacity and costs. A restricted gene panel is required not only to help routine use but, also, to overcome technical challenges. First, cancer generates a chronic physical illness that also affects the vasculature of the circulatory system, making blood drawing from cancer patients challenging and often impairing the quality and quantity of the plasma sample. Additionally, the amount of ctDNA in the plasma and the incidentally present mutation frequencies are often extremely low, which increases the risk of allele drop-out of ultra-rare variants. Moreover, a low on-target ratio implies an increased sequencing load, for which amplicon-based library preparation can provide a solution, but extensive amplification from exceptionally low-input DNA is a source of bias and artefacts. In managing the possible high error rate, a carefully designed NGS library preparation and bioinformatics pipeline can provide the solution. One of the most efficient methods to lower sequencing bias and increased sensitivity is the molecular barcoding technology in NGS-based methods: tagging unique molecular identifiers (UMIs) in the circulating plasma DNA [8]. To provide a reliable solution, here, we developed BC-monitor, a routinely accessible breast cancer-specific NGS-based ctDNA diagnostic tool. We demonstrated the applicability of BC-monitor in a clinical breast cancer setting, including diagnostic tissue samples and serial follow-up plasma samples. From a clinical point of view, we showed that BC-monitor is particularly useful in assisting the precision care of breast cancer patients in both perioperative and metastatic settings.

We determined our assay sensitivity at a 0.5% variant allele frequency, which points to a limitation of the BC-monitor compared to other liquid biopsy assays in the current literature with significantly lower detection limits [23]. Some methods that employ NGS enable the detection of low (<0.1% or ever lower) mutant allele frequencies. However, according to a recently published multi-site cross-platform evaluation of the analytical performance of five industry-leading ctDNA assays, detection becomes unreliable below a 0.5% mutant variant and varies widely between assays, especially when the input material is limited [24].

The gene abnormalities covered by our BC-monitor are important in practice; many of them indicate a poor prognosis (MET, CDH1, TP53, PIK3CA, ESR1, and HER2); the presence of resistance mechanisms (PIK3CA, ESR1, HER2, and FGFR1); or represent druggable targets (PIK3CA, ESR1, HER2, and AKT1) at the same time. According to our experience, the most important benefit of the use of our tool in the metastatic setting was the real-time assessment of those mutations that evolved during disease progression. Hence, the sensitive detection of progression before the clinical signs and symptoms and the selection of a specific therapy among the numerous evidence-based therapy options were often supported by the plasma findings. Furthermore, molecular-targeted therapies, including the HER2 inhibitors, the CDK 4/6 inhibitors, immune checkpoint inhibitors, the PARP inhibitors, and recently, the PI3K inhibitor alpelisib, have become the standard of care; there is a general view that, for the maximum benefit, molecular-targeted agents should be administered early in the disease continuum [25,26]. The ease of use of a liquid biopsy would certainly enhance and alleviate its integration into the forefront of disease management. Additionally, the routine use of a liquid biopsy will surely facilitate patient selection for clinical studies related to drug development [27,28].

The explanation to why we found mutations in the plasma not always identical to the findings in the tumor tissue can be manifold, such as tumor heterogeneity and clonal evolution; nevertheless, serial liquid biopsy results seem to be more helpful for clinical practice than those from the tumor tissue. Regarding circulating tumor marker levels, the relationship between the concentrations of CA 15-3 or CEA and plasma ctDNA was incidental. In fact, according to the guidelines, the use of tumor markers traditionally utilized for monitoring the therapy response nowadays is limited to special situations [29,30]. The information on the fluctuation of genomic alteration levels was suggested to be used both as classical tumor markers indicating the volume of the tumor and as a representation of its clonal evolution [28]. Notably, our study showed that both a-specific therapies such as cytostatic agents and specific target therapies—for example, an mTOR inhibitor in the case of the PIK3CA mutation—did reduce the level of identified mutation in the ctDNA.

In the neoadjuvant cohorts, we found genomic abnormalities in 11/27 pretherapy plasma samples that were cleared out during therapy. A similar rate was reported by Li et al. when ctDNA mutations were detected in 21/44 NCT cases [29]. Our study, in line with other studies, indicated that the longitudinal follow-up of ctDNA mutations outperformed imaging in indicating the response to therapy [29]. Indeed, we also found that serial plasma ctDNA determinations can sensitively predict disease relapse several months earlier than the time of clinical progression and inferior outcome [13,28,31,32]. Several studies pointed to the use of ctDNA technology in post-surgery cases for the detection of minimal residual disease or progression or even for screening high breast cancer risks in healthy individuals [30]. We can also see a potential for BC-monitor in these applications, but we stress the importance of the proper design and evaluation of the chosen diagnostic method.

Traditionally, the intensive follow-up of early breast cancer patients after surgery with conventional diagnostic methods was not recommended due to the lack of benefit of this approach [33]. Nevertheless, owing to advances in our knowledge on the diversity of tumor biology, modern imaging techniques, and novel medical and radiation treatment modalities, it seems possible that, in special cases, the early detection and treatment of progressive diseases improves the outcome [31]. Special situations such as oligometastasis and oligoprogression represent disease entities of low tumor loads, low burdens of mutations with relative molecular homogeneity in the cancer that develops with similar linearity in the primary tumor and metastasis [32]. In these cases, an intensive local and effective systemic therapy results in a cure about 40% of the time [32]. Probably, this field will be the first in which the new technology of liquid biopsy will bring about a paradigm shift in the follow-up strategy [34].

Certainly, there are limitations of the ctDNA tumor diagnostic technology, and the yields of the various approaches and platforms differ. One important challenge is how to interpret negative results, since mutations present in the tumor may remain hidden due to the insufficient release of DNA into the blood, or because the used gene panel does not cover the particular mutation(s) in the tumor. We believe that, since there is a yet-unmet need for utilizing precision molecular diagnostics in oncology care, gaining more knowledge on ctDNA diagnostics and technological advances will reform routine oncology practice in the forthcoming years.

## 5. Conclusions

We conclude that BC-monitor, a breast cancer-specific ctDNA diagnostic approach we developed in this study, has high competence in clarifying preclinical metastases, relapses, and therapeutic resistance. Furthermore, BC-monitor can often pre-indicate disease progression several months earlier than conventional methods. We speculate that the most important benefit of the use of our tool in the metastatic setting is its ability for the real-time assessment of those mutations that evolve during disease progression. Supporting the above, we were able to detect, in a cohort of 45 breast cancer patients, several new pathogenic variants in the follow-up plasma samples that were not detected in the primary tumor and baseline plasma samples. We completed our method development by reducing the targets to the somatic nucleotide variants with the strongest clinical evidence for a poor prognosis in breast cancer and the presence of resistance mechanisms or druggable targets. With the help of this focused gene panel, we were able to find traceable mutations in approximately two-thirds of the patients. We provide evidence that our focused gene panel and bias-reducing bidirectional UMI design for NGS library preparation, in combination with a strict bioinformatic evaluation, resulted in a tool capable of identifying allele variant frequencies below 1% in cell-free plasma DNA. In summary, we suggest that the application of a sensitive real-time ctDNA genotyping tool such as BC-monitor during cancer treatment could guide the treatment strategy and raise therapy effectiveness significantly.

## Figures and Tables

**Table 1:** List of the BC-monitor gene panel with specific hotspots across each gene and positions of the regions of interest according to GRCh38-hg38.

Gene	Exon	Hotspot	Position of Region of Interest
AKT1	2. exon	E17	14:104780165-104780282
CDH1	2. exon	Q23	16:68738248-68738338
EGFR	18. exon	G719	7:55173903-55174022
	19. exon	K745-N756	7:55174737-55174858
	21. exon	L858, L861	7:55191787-55191907
ESR1	7. exon	E380	6:152011675-152011743
	9. exon	S463	6:152094359-152094471
	10. exon	V533-D538	6:152098737-152098851
FGFR1	14. exon	K656	8:38414742-38414820
FGFR4	9. exon	G388	5:177093190-177093300
HER2	8. exon	G309, S310	17:39711902-39712010
	19. exon	L755-D769	17:39723953-39724061
	20. exon	E770-V777	17:39724713-39724788
HRAS	2. exon	G12; G13	11:534221-534315
	3. exon	Q61	11:533852-533947
KRAS	2. exon	G12; G13	12:25245314-25245378
	3. exon	Q61	12:25227309-25227407
MET	14. exon	T1003	7:116771871-116771968
PIK3CA	10. exon	E542; E545	3:179218259-179218324
	21. exon	H1047	3:179234190-179234298
TP53	5-8 exon	coding sequence	17:7673701-7675222/exons/

## Data Availability

All data presented in this study are available upon request (haracska.lajos@brc.hu).

## Supplementary Materials

The following are available online at https://www.mdpi.com/article/10.3390/cancers13143489/s1. Table S1: Coverage information for samples where the whole panel was run, including the healthy samples as well. Each row contains coverage data of the targets, respectively, that belong to a patient or a healthy plasma sample. Table S2: Individual clinical data and molecular findings of cases analyzed having had genomic alterations in the tumor and/or plasma samples: Metastatic breast cancer: MBC, LUMA: luminal A-like, LUMB: luminal B-like, LUMBHER2+: luminal B-like HER2-positive, HER2+: HER2-positive, TNBC: triple-negative, LYM: lymph node metastasis, PLE: pleural metastasis, PUL: pulmonary metastasis, OSS: bone metastasis, HEP: liver metastasis, PER: peritoneal metastasis, CUT: cutaneous metastasis, CR: complete regression, PR: partial regression, SD: stable disease, PD: progressive disease, Doce: docetaxel, Tra: Trastuzumab, Pert: Pertuzumab, Let: Letrozole, Pal: Palbociclib, Gos: goserelin Zol: Zoledronic Acid, Eve: Everolimus, Exe: Exemestane, Pacli: Paclitaxel, Carbo: Carboplatin, Fulv: Fulvestrant, Ribo: Ribociclib, Alpe: Alpelisib, Cape: Capecitabine, Prov: Provera, Ola: Olaparib, and Lapa: Lapatinib. Table S3: Individual clinical data and molecular findings of cases analyzed having had genomic alterations in the tumor and/or plasma samples: Neoadjuvant chemotherapy: NCT, LUMA: luminal A-like, LUMB: luminal B-like, LUMBHER2+: luminal B-like HER2-positive, HER2+: HER2-positive, and TNBC: triple-negative. Denkert tumor regression grade (TRG): 0 = no effect, 1 = resorption and tumor sclerosis, 2 = minimal residual invasive tumor (<5 mm), 3 = residual noninvasive tumor only, and 4 = no tumor detectable. Table S4: Individual clinical data and molecular findings of cases analyzed having had genomic alterations in the tumor and/or plasma samples: Neoadjuvant endocrine therapy: NET, LUMA: luminal A-like, LUMB: luminal B-like, LUMBHER2+: luminal B-like HER2-positive, HER2+: HER2-positive, and TNBC: triple-negative.
